# Strain, cell density, and nutrient condition affect patterns of diurnal vertical migration and superoxide production in a red-tide alga

**DOI:** 10.3389/fcell.2023.1134227

**Published:** 2023-04-13

**Authors:** Tomoyuki Shikata, Saho Kitatsuji, Koki Yuasa

**Affiliations:** ^1^ Fisheries Technology Institute, Japan Fisheries Research and Education Agency, Nagasaki, Goto, Japan; ^2^ Fisheries Technology Institute, Japan Fisheries Research and Education Agency, Hiroshima, Hatsukaichi, Japan

**Keywords:** circadian rhythm, diversity, flagellate, harmful algal bloom, interstrain difference, marine phytoplankton, raphidophyte, swimming

## Abstract

A red tide occurs when cell densities of autotrophic microalgae and some heterotrophic protists increase dramatically and thereby change the color of the sea. Red tides sometimes have negative impacts on human activities, such as fisheries and tourism. Most red-tide flagellates display diurnal vertical migration (DVM) in which cells normally migrate upward during the day and downward at night. This behavior promotes active growth, due to the effective acquisition of nutrients and light, as well as population density increase and cell aggregation. However, the factors and their interactions influencing DVM remain to be clarified, such that no algorithm exists that can precisely simulate the DVM pattern and the development of a red tide in the field. *Chattonella* marina complex (hereafter *Chattonella*) is a representative microalga of harmful red tides and some previous studies has suggested that *Chattonella*’s DVM plays important roles in development of a red tide. *Chattonella* can produce a large amount of superoxide (•O_2_^−^), which is responsible for the regulation of various physiological processes as well as its toxicity against microorganisms and animals. In the present study, we examined the effects of strain, growth phase, cell density, and nutrient deficiency on the pattern of DVM. In addition, we also measured the •O_2_^−^ level in most experiments to assess the relationship between DVM and •O_2_^−^ production. Some strains displayed clear DVM, whereas others aggregated at the surface all day in a fixed condition. Strains’ DVM patterns did not show a relationship with •O_2_^−^ production. Moreover, the DVM became less clear at high cell density and in nitrogen- or phosphorus-depleted conditions. Although a previous study reported that the •O_2_^−^ production rate increased during the light period and decreased during the dark period, regardless of cell density, the diurnal pattern of •O_2_^−^ became less clear at a higher cell density in a *Chattonella* strain used in the present study. Our findings indicate that DVM and •O_2_^−^ production by a *Chattonella* population composed of various strains can change across developmental phases and environmental conditions. This characteristic may produce adaptability in species and increase the chances of a massive population increase.

## 1 Introduction

In marine environments, cell densities of autotrophic microalgae and some heterotrophic protists increase and thereby change the color of the sea—a biological phenomenon known as a red tide. Some red tides negatively impact fisheries and tourism (Sellner et al., 2003), and the impacts of harmful red tides on coastal systems have increased in recent decades (Gobler, 2020). Techniques for mitigating damages in the fish culture industry have been suggested (Kim, 2006; Balaji-Prasath et al., 2022), with precautionary field interventions such as early harvesting, restricting fish feeding, and transport of fish cages into zones free of red tides being the most practical (Kim, 2006). To boost the effectiveness of these precautionary interventions, accurate forecasting of the arrival of a red tide at fishing grounds is necessary. Forecasting based on numerical models is challenging because the densification and expansion of a red tide are influenced by various factors, including biological, chemical, and physical processes (McGillicuddy, 2010; Davidson et al., 2016).

In most cases, the outbreak of a red tide in a marine area requires three steps. First is the appearance of cells in the area due to the transport of cells from other marine areas, growth of surviving cells at quite low density, and excystment. Next is the growth step, in which the cell division rate exceeds the loss rate. The third step is aggregation, which occurs due to physical and biological processes. Various environmental factors influence each of the three steps. As compared to the aggregation process, the growth and life-history stages (including excystment) of red-tide algae have been more thoroughly studied and reported (e.g., Granéli and Turner, 2006).

Most flagellates causing red tides display diurnal vertical migration (DVM) in which cells normally migrate upward during the day and downward at night (Kamykowski, 1995). DVM contributes greatly to the aggregation step (Lai and Yin, 2014) and likely to the growth step as well, as the process offers many ecological benefits to flagellates, such as nutrient acquisition at a wider range of depths, the ability to control the amount of light received for photosynthesis, and escape from predators (Smayda, 1997; Harvey and Menden-Deuer, 2011; Bollens et al., 2012).

Among red-tide flagellates, the DVM pattern follows the photoperiod and appears to be controlled by an endogenous clock (Roenneberg et al., 1989; Shikata et al., 2015), whereas the timing of change in swimming direction and swimming speed differ among species (Kamykowski, 1995; Shikata et al., 2015). Some studies indicated that even if the dominant species is the same across a marine area, DVM patterns can differ among places and days (Katano et al., 2014; Shikata et al., 2020). Previous studies revealed that the DVM pattern is influenced by external environmental factors such as temperature (Heaney and Eppley, 1980), salinity (Jephson et al., 2011), light (Ault, 2000), and nutrient concentrations (Heaney and Eppley, 1980). Based on their different swimming speeds, Bearon et al. (2004) postulated that DVM patterns differ among strains in a flagellate species.

Although numerical simulation models considering physical processes have been developed (Berdalet et al., 2014), no algorithm can precisely simulate DVM patterns of a red-tide algae in the field. This deficiency exists because few studies have assessed both the physical and biological processes underlying DVM, including the physiological responses to various environmental factors, and the biological parameters impacting DVM patterns remain to be clarified for each red tide flagellate.

The raphidophyte *Chattonella marina* complex (hereafter called *Chattonella*) is distributed in warm marine coastal areas around the world and has caused tremendous damage to fisheries (Imai and Yamaguchi, 2012). Reflecting these threats, a large amount of information on *Chattonella* has been gathered, particularly with regard to its growth physiology, life cycle, and interaction with other microorganisms. Field data collected before and after *Chattonella* red tides are also available for designing laboratory experiments and validating numerical models using biological parameters. In addition, studies on the DVM of *Chattonella* have been conducted as well. A field experiment using a mesocosm revealed that *Chattonella* could reach a depth of 7.5 m during DVM, and its migration speed was 0.8 m h^−l^ both upward and downward (Watanabe et al., 1995). *Chattonella* can sense weak UV and blue light, allowing it to cue its DVM to the day–night cycle (Shikata et al., 2013). Negative phototaxis was observed under a monochromatic light with UV and blue components, but the response disappeared by mixing light with longer wavelengths (Shikata et al., 2016). Although one report has noted chemotaxis toward inorganic phosphate (Ikegami et al., 1995), we also observed a clear DVM in *Chattonella* without a vertical gradient of phosphate concentration (Shikata et al., 2015). Therefore, the DVM pattern may be regulated mainly by the endogenous clock that synchronizes the photoperiod and switches between positive and negative geotaxis in *Chattonella*. However, low salinity in the upper layers arrests the upward migration of *Chattonella* (Katano et al., 2014; Shikata et al., 2014), and a field study of DVM showed that cell density in the water column might also affect DVM (Sakaguchi et al., 2017). These findings indicate that there are also factors inhibiting the DVM pattern regulated by the endogenous clock.

*Chattonella* can produce a large amount of superoxide (•O_2_^−^) in the cell membrane and excrete it from the cell. This •O_2_^−^ and hydrogen peroxide has antimicrobial activity and is a candidate toxin related to fish mortality (Oda et al., 1992), as the level of •O_2_^−^ is highly correlated with toxicity to aquacultured fishes in seawater that contains *Chattonella* cells (Shikata et al., 2021). Ichthyotoxicity can disappear within a short period and only live *Chattonella* cells can kill fishes; ruptured cells and culture supernatant have no toxicity to fish (Matsusato and Kobayashi, 1974; Ishimatsu et al., 1996; Shen et al., 2010). These characteristics correspond with chemical unstableness of •O_2_^−^. The amount of •O_2_^−^ production is influenced by nutrient and light intensity conditions; nutrient deficiency and strong light can increase the production (Yuasa et al., 2020a; b). Moreover, *Chattonella* exhibits different •O_2_^−^ production rates in the light and dark periods (Kim et al., 2005; Yuasa et al., 2020a). These results suggest that •O_2_^−^ may be responsible for regulating various physiological processes with circadian rhythms.

In the present study, we examined the effects of strain, growth phase, cell density, and nutrient deficiency—factors scarcely addressed thus far—on the DVM pattern in *Chattonella*. In most experiments, we also simultaneously measured the •O_2_^−^ level to investigate the potential synchronous patterns of DVM and •O_2_^−^ production in this flagellate species.

## 2 Materials and methods

### 2.1 Culture of strains

Five clonal strains of *Chattonella* were used in this study. The dates and locations for the isolation of these strains are listed in Table 1. Two strains were axenic, and the others were not. The strains were subcultured in 50-mL Erlenmeyer flasks containing 25 mL of modified SWM-3 medium (Shikata et al., 2011) with a salinity of 32 psu at 25°C under 150 μmol photons m^−2^ s^−1^ of white fluorescent irradiation on a 12:12-h light:dark cycle (light period, 06:00 to 18:00 local time [LT]). The photosynthetic photon flux density in the incubator was measured with a Quantum Scalar Laboratory PPFD sensor (QSL-2101, Biospherical Instruments Inc., San Diego, CA, United States).

**TABLE 1:** *Chattonella* strains used in the present study. All strains were isolated from Japanese coastal areas. In cell shape, the length:width ratio of *Chattonella antiqua*-like cells are larger than *Chattonella marina*-like cells (Shikata et al., 2021).

Strain name	Year collected	Origin	Contamination status	Shape^1)^	Ichthyotoxicity^1)^	References
NIES-1	1978	Harima-nada	Axenic	*C. antiqua*-like	High	1)
OP27	2010	Ariake Sea	Xenic	*C. antiqua*-like^2)^	High	3)
Ago03	2013	Ago Bay	Xenic	*C. marina*-like	High	1)
Ago04	2013	Ago Bay	Xenic	*C. marina*-like	Low	1)
3KGY	2010	Yatsushiro Sea	Axenic	*C. antiqua*-like	Medium	1)

^1)^ Shikata et al. (2021); ^2)^ Akita et al. (2022); ^3)^ Wada et al. (2018).

### 2.2 Monitoring DVM with a cylindrical aquarium

A cylindrical aquarium was used as a culture vessel to monitor DVM (Shikata et al., 2014). The aquarium (inner diameter, 5 cm; height, 90 cm) was made of acrylic, with nine sampling ports (diameter, 13 mm) at intervals of 10 cm, starting from the bottom. Each port was plugged with a silicone-rubber stopper. The aquarium was placed in a temperature-controlled room (25°C) and capped with a disc-shaped LED lamp (daylight color; INREDA, Spot-light LED, Inter IKEA Systems B.V., The Netherland). The light intensity at the surface was 250 µmol photons m^−2^ s^−1^.

On the day prior to each experiment, we added modified SWM-3 medium and a cell suspension at 17:00–18:00 LT. The total volume of the medium and the cell suspension was adjusted to 1.6 L, and the final water depth in the aquarium was about 85 cm. Each experiment was initiated at 09:00 LT, and at 3-h intervals 5 mL of the cell suspension was sampled from the water surface or from each sampling port. Then a syringe and a needle were used to refill the aquarium from the surface and bottom sampling ports with 25 mL of fresh medium.

Yamaguchi et al. (1991) reported that the cell densities of *Chattonella* are positively correlated with *in vivo* fluorescence. Therefore, to determine the vertical distribution of cells, we measured the *in vivo* fluorescence (Brand et al., 1981) of the sample at each depth by using a fluorometer (Model 10-AU; Turner Designs, Sunnyvale, CA, United States). Cell densities were calculated using a standard curve for each strain, although only cell densities at the surface and bottom were counted under a light microscope (Eclipse TS-100; Nikon Co., Tokyo, Japan). For sample collection during the dark period, we used a red LED flashlight (peak wavelength, 630 nm) for illumination, because red light has little effect on the DVM pattern of *Chattonella* (Shikata et al., 2013).

### 2.3 Monitoring DVM with a digital camera system

The system described by Shikata et al. (2016) was also used for monitoring DVM. A rectangular acrylic chamber (base: 1 cm wide × 5 cm long) containing 25 mL of cell suspension (depth, 5 cm) was held by a clamp. Each sample was illuminated by a white LED from above; the photon flux density at the water surface was adjusted to 150 µmol photons m^−2^ s^−1^.

A monochromatic, digital, charge-coupled device camera (Pixelfly, PCO AG, Kelheim, Germany) was used to photograph the sample from the side. A long pass filter (cut off wavelength of <800 nm; LX-903, Mitsubishi Layon Co. Ltd., Tokyo, Japan) was mounted in front of the camera lens. The samples were illuminated by an infrared LED (peak wavelength, 850 nm; Pi Photonics Inc., Shizuoka, Japan) from behind, and the camera captured the light scattered by the cells. Each sample was time-lapse photographed every 15 min for 66 h (from day 0 at 18:00 to day 2 at 12:00 LT) to obtain raw data for determining the DVM. The devices that composed the system were operated in a temperature-controlled (25°C) dark room.

In the digital images, the cell density in each area is roughly reflected by its brightness. Since the brightness of a pixel in the 8-bit image is represented by 256 (2^8^) steps of gray values, we first read the gray values of pixels at respective areas of cell suspension using ImageJ software (http://rsbweb.nih.gov/ij/) to evaluate vertical profiles of the cells. The surface accumulation ratios were then calculated as the common logarithm of the ratio of the average of gray values in the surface layer (0–0.75 cm) and the bottom layer (2.50–3.00 cm). Because *Chattonella* generally migrated between the surface and bottom under most experimental light conditions, the time series of surface accumulation ratios was used to represent the DVM pattern and cell accumulation. High and low ratios were recorded when most cells were swimming upward and downward, respectively.

### 2.4 •O_2_^−^ detection

•O_2_^−^ was detected using the chemiluminescence reagent L-012 (FUJIFILM Wako Pure Chemical Corp., Osaka, Japan) (Nishinaka et al., 1993). The resultant chemiluminescence was monitored for 30 s using a luminometer (AB-2270 Luminescencer Octa, ATTO Corp., Tokyo, Japan). The •O_2_^−^ levels in cultures were calculated by subtracting the chemiluminescence measured in the presence of 200 U of bovine superoxide dismutase (SOD; FUJIFILM Wako Pure Chemical Corp.) from the measured chemiluminescence.

### 2.5 Statistical analyses

The data were tested for homogeneity of variances using Levene’s test. When variances were homogeneous, we used one-way analysis of variance and multiple comparisons with Tukey’s honestly significant difference (HSD) test to assess the differences in *Chattonella* biological parameters among treatments. Data not showing homogeneous variances were log-transformed, and a Levene’s test was performed once again. When the variances were not homogeneous even after log-transformation, we used the Dunnet T3 test. All analyses were performed with IBM SPSS Statistics Desktop Version 19.0 for Windows (IBM Japan, Tokyo, Japan), with *p* < 0.05 indicating a significant difference.

## 3 Results

### 3.1 Patterns of DVM and superoxide production in different *Chattonella* strains

We observed DVM in four strains of *Chattonella* (NIES-1, OP27, Ago03, and Ago04) using the cylindrical aquarium. Approximately equal amounts of cells (∼3.0 × 10^5^ cells) at the late logarithmic growth phase (late log phase; 1.5–3.0 × 10^4^ cells mL^−1^) were injected into the aquarium. The vertical cell distribution at each time was represented by the cell accumulation ratio (%), calculated as the cell abundance at a sampling depth layer divided by the sum of cell abundances at all sampling depth layers. Average cell densities in the water column over the observation periods ranged from 2.5 × 10^2^ to 6.1 × 10^2^ cells mL^−1^ in the four strains (Supplementary Figure S1). It was a common trend among strains that average cell abundance in the water column gradually decreased until the middle of the dark period and then recovered. A decline in the average cell abundance might have been caused by the aggregation of cells among sampling ports, whereas an increase might have been caused by sampling and cell division, because most *Chattonella* cells divide during the latter half of the dark period (Nemoto and Furuya, 1985).

**FIGURE 1:**
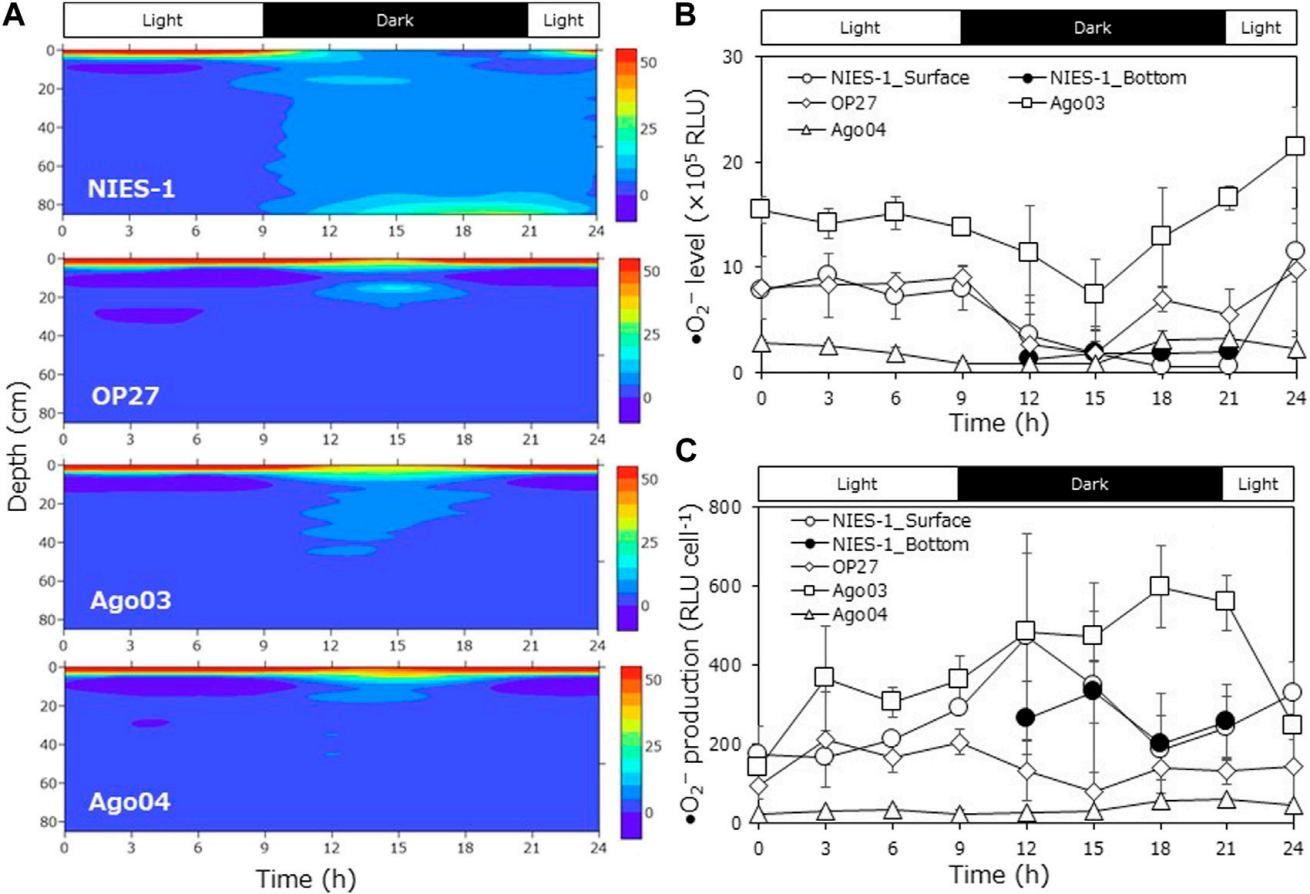
Diurnal vertical migration of different *Chattonella* strains in the cylindrical aquarium. **(A)** Time-course variations in the cell accumulation percentage at each depth layer (average of triplicate measurements), **(B)** time-course variations in superoxide (•O_2_^−^) level (relative luminescence units: RLU), and **(C)** •O_2_^−^ production (•O_2_^−^ level per cell) of *Chattonella* strains at the surface or bottom layer (only dark periods of NIES-1) in the cylindrical aquarium. •O_2_^−^ level was measured by a chemiluminescence method. Each bar represents the mean ± SD of three biological replicates.

The strain NIES-1 displayed clear DVM (Figure 1A). Most cells started downward migration at the transitional period from light to dark (at 9 h from the start time), and a portion of the cells reached the bottom during the dark period. Maximum cell accumulation ratios at the bottom were 34.6% ± 11.7% (average ±SD). Thereafter, cells rapidly migrated to the surface at the transitional period from dark to light (at 21–24 h). The cell accumulation ratio at the surface varied from 4.7% ± 1.6% to 82.0% ± 2.0% through the experimental period.

In the other strains, most cells also accumulated at the surface during the light period (Figure 1A). However, most cells continued to aggregate at the surface and scarcely reached the bottom during the dark period. Maximum cell accumulation ratios at the bottom were 2.8% ± 2.3% in OP27, 3.2% ± 1.9% in Ago03, and 0.7% ± 0.2% in Ago04; these values were significantly lower than that of NIES-1 (Turkey test; *p* < 0.05). The cell accumulation ratio at the surface ranged from 36.3% ± 1.5% to 98.6% ± 0.38% in OP27, from 36.0% ± 15.5% to 96.8% ± 0.6% in Ago03, and from 51.3% ± 6.3% to 98.2% ± 0.8% in Ago04 during the experimental period.

Levels of •O_2_^−^ (relative luminescence units: RLU) and •O_2_^−^ production (RLU cell^−1^) both differed among strains (Figures 1B,C). Although levels of •O_2_^−^ at the surface decreased during the dark period and then increased during the light period in all strains, the maximum •O_2_^−^ production in Ago04 was significantly lower than rates in other strains (Turkey test after log-transformation; *p* < 0.05). In NIES-1 and OP27, •O_2_^−^ production increased during the light period, peaked around the beginning of the dark period (9–12 h), and then decreased at the start of the light period (21 h). In Ago03 and Ago04, however, •O_2_^−^ production increased during the dark period and peaked around the beginning of the light period (18–21 h). We also measured the •O_2_^−^ production level at the bottom for only NIES-1 during a part of the dark period, but the value was similar to that at the surface.

### 3.2 Patterns of DVM and superoxide production in different growth phases and cell densities

We investigated the effects of culture growth phase and cell density on the DVM pattern within the cylindrical aquarium. The strain 4KGY was used in this experiment. Cultures at the early log phase (4.6 × 10^3^ cells mL^−1^) and late log phase (2.6 × 10^4^ cells mL^−1^) were prepared. The culture at the early log phase was injected into the aquarium. Average cell density in the water column over the observation period was 6.3 × 10^2^ cells mL^−1^. Next, cultures in the late log phase were inoculated into separate aquaria at three different doses (low cell density, LD; medium cell density, MD; high cell density, HD). Average cell densities in the water column in LD, MD, and HD over the observation period was 2.9 × 10^2^ cells mL^−1^, 1.3 × 10^3^ cells mL^−1^, and 2.1 × 10^4^ cells mL^−1^, respectively (Supplementary Figure S2). As in the interstrain comparison experiments, the average cell abundance in the water column gradually decreased until the middle of the dark period and then recovered (Supplementary Figure S2). In the HD treatment, a portion of the cells aggregated at the bottom just after cell inoculation, and we could not sample the aggregate due to its stickiness. The average cell density in the water column did not decrease during the experimental period, meaning the aggregate scarcely increased (Supplementary Figure S2).

**FIGURE 2:**
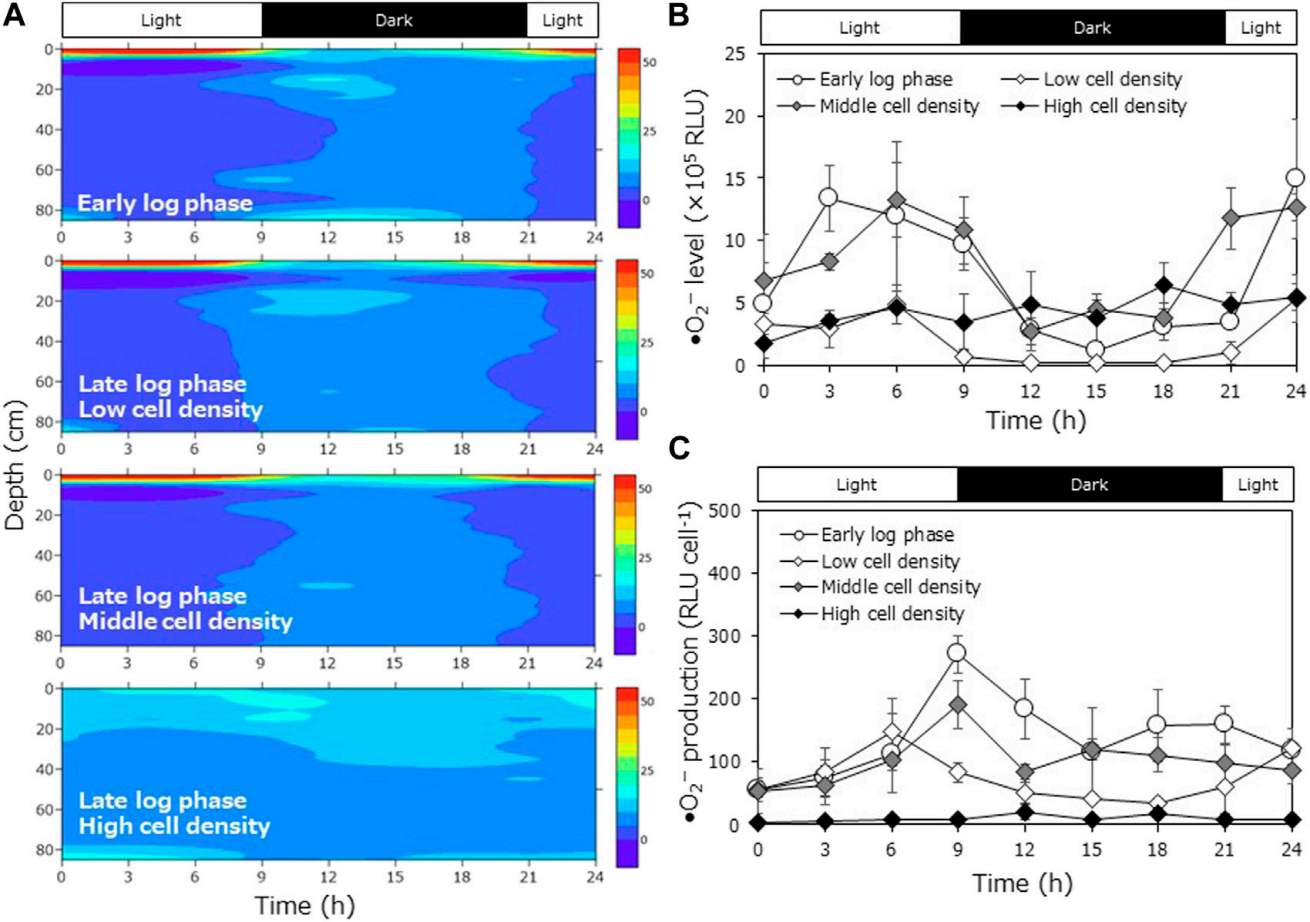
Diurnal vertical migration of *Chattonella* at different growth phases and cell densities in the cylindrical aquarium. **(A)** Time-course variations in the cell accumulation percentage at each depth (average of triplicate measurements), **(B)** time-course variations in superoxide (•O_2_^−^) level (relative luminescence units: RLU), and **(C)** •O_2_^−^ production (•O_2_^−^ level per cell) of *Chattonella* at the surface or bottom layer in the cylinder aquarium. •O_2_^−^ level was measured by a chemiluminescence method. Each bar represents the mean ± SD of three biological replicates.

Cells in the early log phase displayed clear DVM (Figure 2A). Most cells started downward migration at the transitional period from light to dark (at 9 h), and a portion of the cells reached the bottom during the dark period. The maximum cell accumulation ratio at the bottom during the dark periods was 24.3% ± 12.0%. The surface cell accumulation ratio ranged from 7.7% ± 1.5% to 91.0% ± 1.9% throughout the experimental period. As with early log phase culture, cells at the late log phase at the low or medium cell density also migrated downward during the dark period. However, the maximum cell accumulation ratio at the bottom during the dark period was lower than that of the early log phase culture: 12.1% ± 3.5% at LD and 7.3% ± 1.9% at MD, although no statistically significant differences were detected (Turkey test; *p* = 0.304 for LD, *p* = 0.110 for MD). The cells did not display clear DVM at HD; cells accumulated within the upper half of the aquarium or near the bottom all day. At HD, the maximum cell accumulation ratio at the surface over the experimental period was significantly lower than in the early growth phase culture and at the other cell densities (Turkey test; *p* < 0.05).

We measured the •O_2_^−^ level at the depths where cells accumulated most (surface or bottom). At LD and MD, levels of •O_2_^−^ decreased during the dark period and then increased during the light period (Figure 2B). •O_2_^−^ production increased during the light period and peaked during the light period (6–9 h; Figure 2C). At HD, clear rhythms of •O_2_^−^ level and •O_2_^−^ production were not observed. The •O_2_^−^ production was very low all day, and the maximum value was significantly lower than those in other conditions (Dunnet T3 after log-transformation; *p* < 0.05).

### 3.3 DVM patterns and superoxide production under different nutrient conditions

We investigated the effects of nutrient deficiency on the DVM pattern using the digital camera system. Although it is difficult to completely remove nutrients from the medium based on natural seawater, the growth of *Chattonella* strains in artificial medium is not good. Therefore, we adopted the digital camera system, which requires no exchange of medium, in this experiment. Axenic strain 4KGY was incubated in medium lacking NaNO_3_ (N-depleted medium), lacking NaH_2_PO_4_·2H_2_O (P-depleted medium), or containing all components (complete medium) for 5 days before the observation of DVM. The cell densities were ∼2.0 × 10^4^ cells mL^−1^ at the start of the experiment.

**FIGURE 3:**
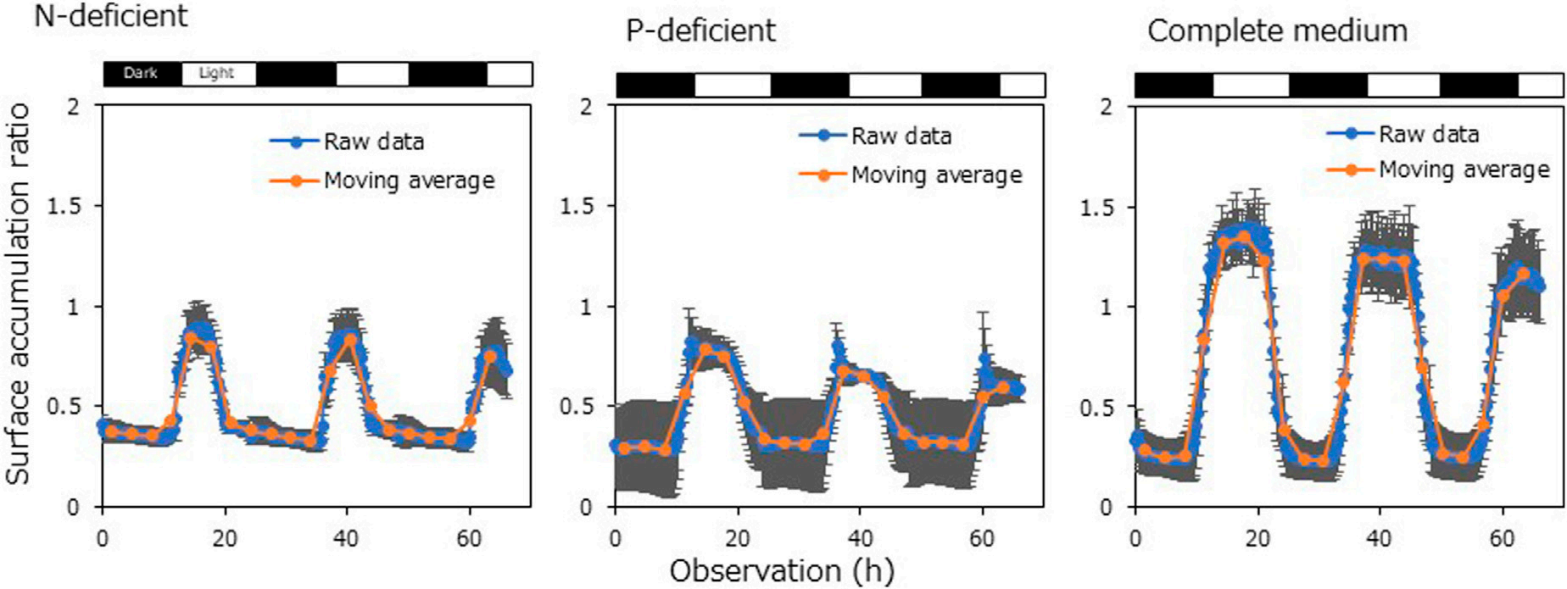
Diurnal vertical migrations of *Chattonella* at different degrees of nutrient deficiency observed by the digital camera system. Raw data and moving average at 3-h intervals are shown. Each bar represents the mean ± SD of four biological replicates.

**FIGURE 4:**
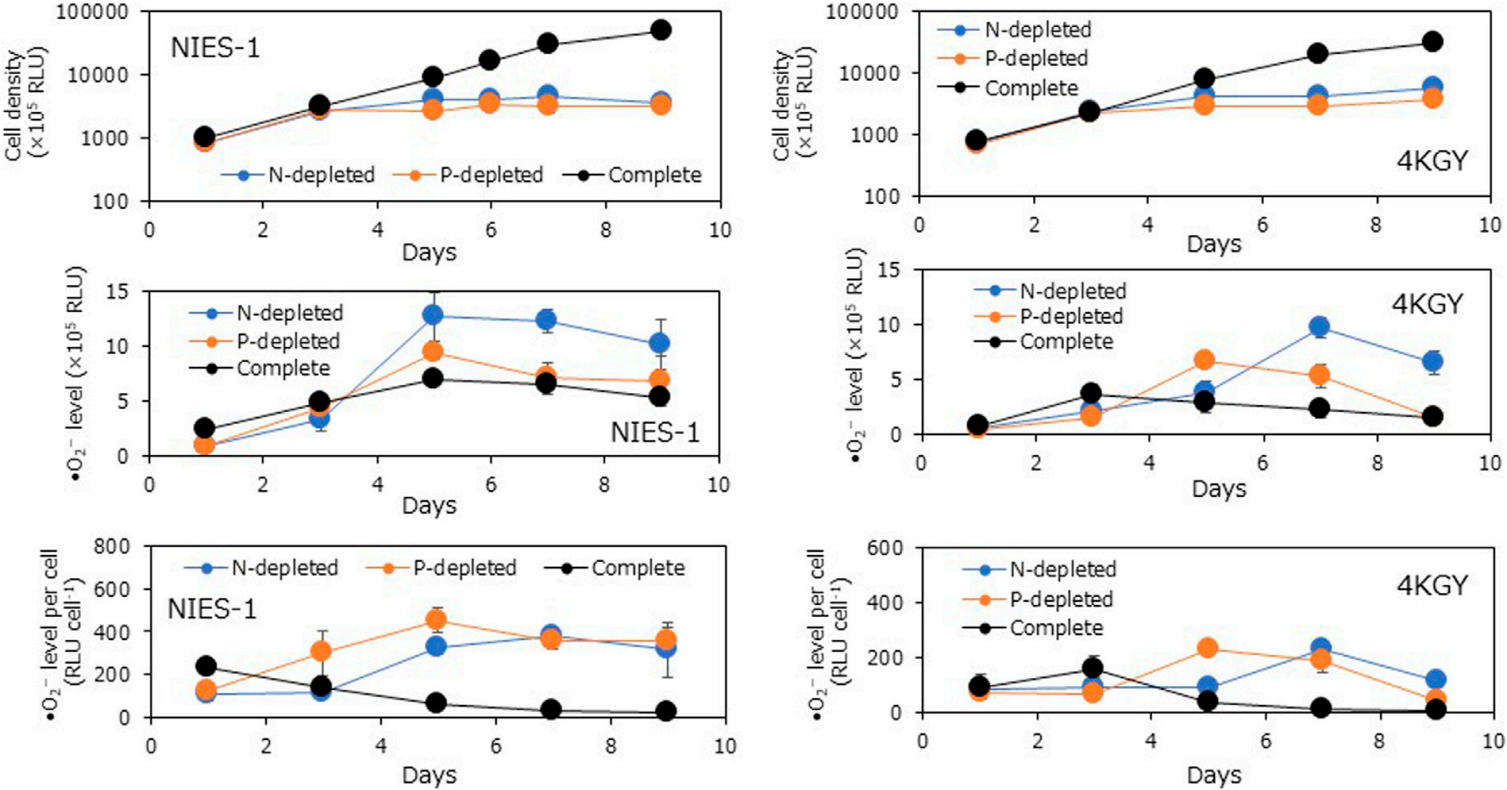
Time-course variations in cell density, superoxide (•O_2_^−^) level (relative luminescence units: RLU), and •O_2_^−^ production (•O_2_^−^ level per cell) of two *Chattonella* strains at different degrees of nutrient deficiency. •O_2_^−^ level was measured by a chemiluminescence method. Each bar represents the mean ± SD of three biological replicates.

Cells displayed DVM under all nutrient conditions, but surface accumulation ratios under nutrient-depleted conditions were lower than in the complete medium treatment during the light period (Figure 3). There were significant differences in the maximum moving averages of the surface accumulation ratio measured at 3-h intervals between the nutrient-depleted media and the complete medium on both days (Dunnett T3; *p* < 0.05). Moreover, to investigate the effects of nutrients on •O_2_^−^ production in different *Chattonella* strains, we incubated axenic strains NIES-1 and 4KGY in 50-mL Erlenmeyer flasks containing 25 mL of N-depleted medium, P-depleted medium, or complete medium and tracked the cell density and •O_2_^−^ level for 9 days. Conditions of temperature and light were the same as in the subculture. Cells increased throughout the experimental period in the complete medium, but increases in cell densities stopped from day 5–7 under the nutrient-depleted conditions (Figure 4). The •O_2_^−^ level in NIES-1 and 4KGY cultures peaked from days 5–7 under all conditions, but the maximum values under nutrient-depleted conditions were significantly higher than those in complete medium and that under N-depleted condition was significantly higher than that in P-depleted condition (Dunnet T3; *p* < 0.05). Production of •O_2_^−^ by the two strains rapidly increased on day 5 under nutrient-depleted conditions, whereas it gradually decreased from day 1 in the complete medium. The maximum •O_2_^−^ production by NIES-1 under nutrient-depleted conditions was significantly higher than that in complete medium (Dunnet T3; *p* < 0.05). For 4KGY, there were not statistically significant differences in the maximum •O_2_^−^ production among nutrient conditions, but that under nutrient-depleted conditions was significantly higher than that in complete medium on day 5 (Turkey test; *p* < 0.05) and day 7 (Dunnet T3; *p* < 0.05). The maximum •O_2_^−^ level and •O_2_^−^ production of NIES-1 were significantly higher than those of 4KGY under each condition (Dunnet T3 after log-transformation; *p* < 0.05).

## 4 Discussion

Among investigations conducted under quiet conditions in enclosed coastal areas or in a mesocosm, there were differences in the depth reached by a *Chattonella* red tide at night (Watanabe et al., 1995; Araki et al., 2013; Sakurada et al., 2013; Katano et al., 2014; Sakaguchi et al., 2017). In previous laboratory studies using *Chattonella* strains NIES-1 and 3KGY, most *Chattonella* cells migrated to the surface of a vessel during the light periods and to the bottom during the dark periods (Kim et al., 2005; Shikata et al., 2014). Before the present study, however, we viewed flasks including *Chattonella* strains during the dark periods and found that some strains aggregated at the surface of the medium even during the night. The present study showed that some strains display clear downward migration during the dark periods, whereas others scarcely migrate under the same condition. Strains Ago03 and Ago04 were isolated from the same marine area during different years, and both displayed weak downward migration. Strains 4KGY used in the present study and 3KGY used in Shikata et al. (2014) were isolated from the same marine area on the same day. Compared to 4KGY (early log phase), 3KGY has higher surface accumulation ratios during the dark periods and accumulates at the bottom during the light periods in the same experimental setup, indicating that DVM in 3KGY has more entrainability. Although *Chattonella* shows variations in cell shape and toxicity among strains (Demura et al., 2009; Shikata et al., 2021), the relationship between their phenotypes (Table 1) and DVM pattern remains obscure. Our findings indicate that the strain composition of a *Chattonella* population may influence the vertical cell distribution at each time point in the field, although it is unclear what produced the interstrain differences in the DVM pattern.

Interstrain variation in the DVM pattern may contribute to species survival by decreasing the risk that all cells are exposed to a harmful environment at a certain depth. However, if only strains with a poor ability of downward migration survive, it is unclear whether the population could recover the ability. Moreover, the large diversity in DVM can inhibit densification of cells, leading to poorer competition with other microorganisms, such as antibiotic ability (Oda et al., 1992) and toxicity against other phytoplankters (Qiu et al., 2011; Fernández-Herrera et al., 2016). Therefore, the strain composition of a *Chattonella* population may characterize the population dynamics in each marine area.

It is difficult to incubate some *Chattonella* strains at large volumes (>200 mL) in normal vessels such as an Erlenmeyer flask for days, although the reasons are unclear. Surprisingly, the present study revealed that strains OP27, Ago03, and Ago04, which can grow in large-scale incubation, displayed little downward migration. It is impossible to resuspend a cell pellet made by centrifugation of a *Chattonella* culture because the cells strongly adhere to each other at high cell density (Jenkinson et al., 2007). Although bioconvection occurs at the surface and mitigates densification during periods of upward migration, bioconvection does not occur at the bottom during periods of downward movement. Therefore, success in the large-scale incubation of *Chattonella* may be closely related to the ability of a strain to migrate downward.

Sakaguchi et al. (2017) reported that a red tide of *Chattonella* displays clear DVM when the average cell density is on the order of 10^3^ cells mL^−1^ in the water column, whereas downward migration is scarcely observed when the average cell density is on the order of 10^4^ cells mL^−1^ and nutrient concentrations are sufficient for *Chattonella* growth. Likewise, in the present study *Chattonella* cells scarcely displayed DVM when the average cell density in the water column (volume: 1.6 L) was more than 10^4^ cells mL^−1^ (late log phase in Figure 2). Although the maximum cell density of *Chattonella* was normally on the order of 10^4^ cells mL^−1^ even when the other conditions were suitable for growth, the growth phase at the time of the inoculation scarcely affected DVM patterns. Therefore, densification may hamper various physiological activities related to downward migration and growth, although the mechanism remains to be clarified. When the sample volume was small (25 mL), however, clear DVM was observed even on the order of 10^4^ cells mL^−1^ (complete medium in Figure 3). In *Chattonella*, at greater incubation volume, the growth rate and maximum cell yield were lower. Thus, the effects of densification on physiological states may depend on the incubation volume in laboratory studies.

In various flagellate species, the depletion of nitrogen or phosphorus activates or inhibits DVM (Heaney and Eppley, 1980; Yuasa et al., 2018). We observed that the deficiency of nitrogen or phosphorus inhibited DVM in *Chattonella* with two axenic strains. Although the red-tide dinoflagellate *Karenia mikimotoi* aggregates at the surface all day under nitrogen-depleted conditions (Yuasa et al., 2018), we found that *Chattonella* sank more easily in nitrogen- or phosphorus-depleted conditions. In a Swedish fjord, the amount of *Chattonella* cells peaked in sediment traps deployed in deep layers (15 and 30 m depths) when a red tide of *Chattonella* was declining and nitrate concentrations in the surface layer (0–10 m depth) were low (Waite and Lindahl, 2006). In addition, DVM ceases under continuous darkness, whereas it persists under continuous red light, which acts on photosynthesis but scarcely shifts the phase of the DVM pattern (Shikata et al., 2015). Therefore, swimming would require energy from photosynthesis, which in turn requires light and nutrients, in *Chattonella*.

Kim et al. (2005) examined the relationship between the production of reactive oxygen species and DVM using the *Chattonella* strain NIES-1. They observed DVM similar to that recorded in the present study and found that •O_2_^−−^ production was high during the light period and that it decreased during the dark period in cell-aggregated layers (surface or bottom). Yuasa et al. (2020a) observed a similar diurnal pattern in •O_2_^−^ production in the strain 4KGY. The present study also revealed a similar pattern of •O_2_^−^ production in the strain OP27, but the DVM pattern of strains Ago03 and Ago04 was different from that of the other strains, with •O_2_^−^ production increasing during the dark period. Interestingly, we noticed that characteristics of the cellular •O_2_^−^ production pattern may be categorized by cell shape in *Chattonella* strains, as noted in strains with a *Chattonella antiqua–*like shape (NIES-1, 4KGY, OP27) and strains with a *C. marina–*like shape (Ago03, Ago04); the amount of •O_2_^−^ production, however, is not categorized by cell shape among strains (Shikata et al., 2021). These findings imply that the DVM pattern may be independent of the •O_2_^−^ production pattern because only the latter was categorized by cell shape.

Based on experiments using the strain NIES-1, Kim et al. (2004) suggested that the growth phase (or cell density) can affect the amount of •O_2_^−^ production but has little effect on the diurnal pattern of •O_2_^−^ production. The •O_2_^−^ production rate had a maximum value during the exponential growth phase (low cell density) and subsequently decreased in the stationary phase (high cell density), but •O_2_^−^ production increased during the light period and decreased during the dark period regardless of growth phases. However, the present study using strain 4KGY showed that the diurnal pattern of •O_2_^−^ production was less clear at higher cell density (stationary phase).

Kim et al. (2004) reported that •O_2_^−^ production was lower when nitrogen and phosphorus concentrations were low than when these nutrients were fully supplied in strain NIES-1. On the other hand, Yuasa et al. (2020a) reported that •O_2_^−^ production is stimulated under nitrogen- or phosphorus-deficient conditions in the 4KGY strain. Therefore, the response of •O_2_^−^ production to nutrient deficiency appears to depend on the strain. According to the reverification result in the present study, however, •O_2_^−^ production was stimulated under nitrogen- or phosphorus-deficient conditions in both NIES-1 and 4KGY. Previous studies have suggested that the reducing power required for •O_2_^−^ production in the cell membrane may originate from photosynthesis, based on inhibition of •O_2_^−^ production by the administration of a photosynthetic inhibitor during the light period as well as the increase in •O_2_^−^ production during the light period (Marshall et al., 2002; Yuasa et al., 2020b). Yuasa et al. (2020a) have reported that •O_2_^−^ production under nitrogen- or phosphorus-deficient conditions increase during the dark period and the extracellular levels of •O_2_^−^ under nutrient-deficient conditions are unaffected by the presence of an inhibitor of photosynthetic electron transport—indicating that pathways of •O_2_^−^ production other than photosynthesis exist. However, the present study revealed that diurnal patterns of •O_2_^−^ production differ among strains. Thus, there may be differences among strains with regard to the degree of dependence on each production pathway and sensitivity to environmental conditions such as cell density and nutrient availability.

In conclusion, our findings indicate that strain, cell density, and nutrient deficiency are factors that affect the DVM pattern and •O_2_^−^ production in *Chattonella*. Moreover, our results suggest that the diurnal patterns of vertical migration and •O_2_^−^ production may be independently regulated, indicating that a *Chattonella* population can have cells with numerous combinations of DVM and •O_2_^−^ production patterns. A great diversity of DVM and •O_2_^−^ production in a population should increase the chances of acquiring nutrients and light and successfully competing against other microorganisms in the water column. Quantitative analyses using numerical models based on the information obtained in the present study are important future tasks.

## Data Availability

The original contributions presented in the study are included in the article/Supplementary Material, further inquiries can be directed to the corresponding author.
