# Supplementary figures and images for: Strain, cell density, and nutrient condition affect patterns of diurnal vertical migration and superoxide production in a red-tide alga
Source: Front Cell Dev Biol. 2023 Apr 13;11:1134227. doi: 10.3389/fcell.2023.1134227 (PMC10133536; doi:10.3389/fcell.2023.1134227)

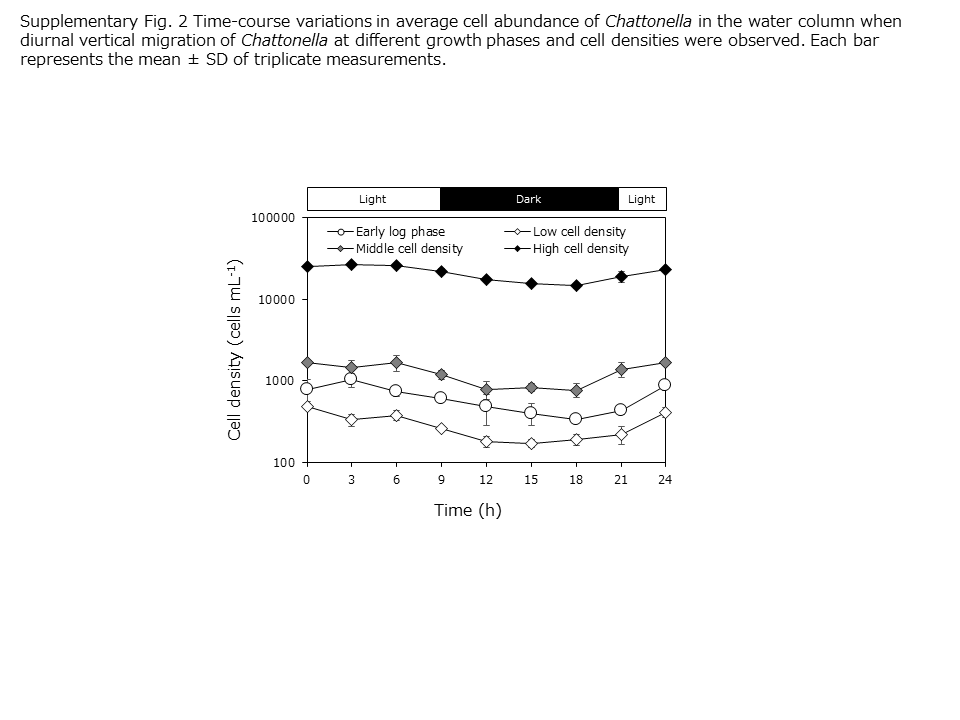

Supplement: Supplementary file 1 [file Image2.tif]

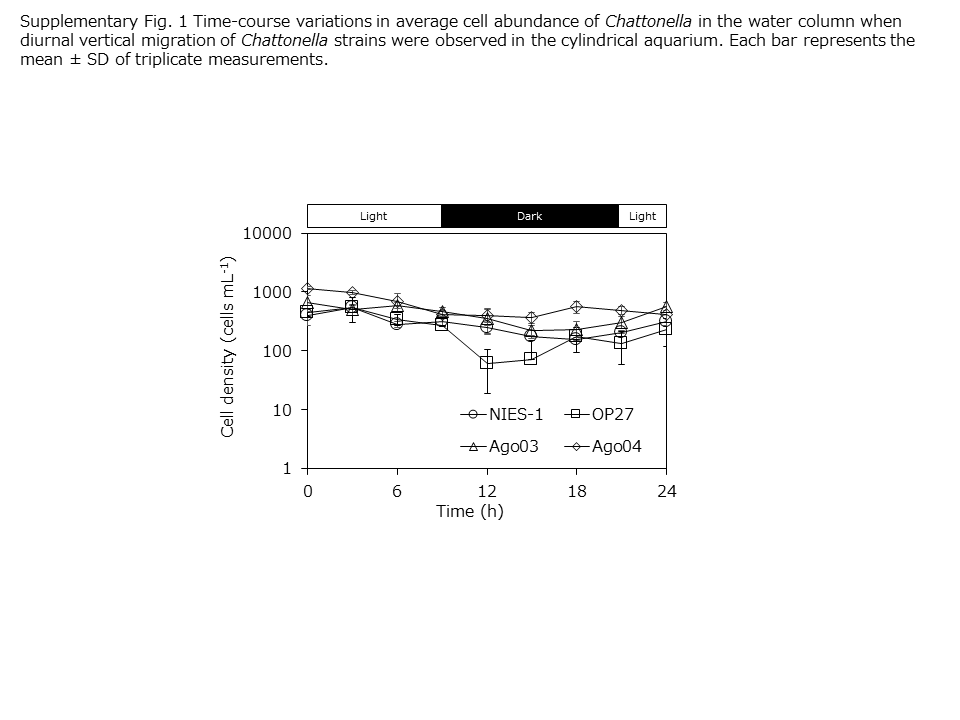

Supplement: Supplementary file 2 [file Image1.tif]
